# Doppler Sonography Confirmation in Patients Showing Calcified Carotid Artery Atheroma in Panoramic Radiography and Evaluation of Related Risk Factors

**DOI:** 10.5681/joddd.2012.002

**Published:** 2012-03-13

**Authors:** Mahrokh Imanimoghaddam, Mohammad Rah Rooh, Elahe Mahmoudi Hashemi, Abbas Javadzade Blouri

**Affiliations:** ^1^Dental Research Center, Mashhad University of Medical Sciences, Mashhad, Iran; ^2^Associate Professor, Department of Oral and Maxillofacial Radiology, School of Dentistry, Mashhad University of Medical Sciences, Mashhad, Iran; ^3^Associate Professor, Department of Radiology, School of Medicine, Mashhad University of Medical Sciences, Mashhad, Iran; Post-graduate Student, Department of Oral and Maxillofacial Radiology, School of Dentistry, Mashhad University of Medical Sciences, Mashhad, Iran ^4^; ^5^Associate Professor, Department of Oral Medicine, School of Dentistry, Mashhad University of Medical Sciences, Mashhad, Iran

**Keywords:** Calcified carotid artery atheroma, Doppler sonography, panoramic radiography

## Abstract

**Background and aims:**

The purpose of this study was to identify patients at the risk of cerebrovascular attack (CVA) by detecting calcified carotid artery atheroma (CCAA) in panoramic radiography and evaluating their risk factors.

**Materials and methods:**

A total of 960 panoramic radiographs of patients above 40 years old were evaluated. Doppler Sonography (DS) was performed for patients who showed calcified carotid artery atheroma (CCAA) in panoramic radiogra-phy in order to determine the presence of CCAA and the degree of stenosis. Cardiovascular risk factors in both groups of patients with CCAA (12 subjects) and without CCAA (3 subjects) were compared using a questionnaire filled out by the patients. Statistical analysis including Fisher and independent t-test applied for data analysis.

**Results:**

Fifteen patients (30 sides) showed calcification in their panoramic radiographs, and underwent DS which revealed CCAA in 16 sides (12 patients). Two patients (13.33%) showed stenosis greater than 70%. Among the risk factors, only age showed a significant association with the occurrence of carotid calcified atheroma (P=0.026).

**Conclusion:**

Considering the results, dentists should refer especially elderly patients with radiographically identified atheromas for further examinations, as asymptomatic CCAA might be associated with high degrees of stenosis.

## Introduction

Carotid atheroma is an atherosclerotic process occurring along the lumen walls of the common carotid artery near its bifurcation. Pieces of the atheroma may ulcerate and become detached to form an embolus that can occlude smaller intracerebral arteries, causing stroke.^1^ Approximately 80% of the strokes is of the ischemic type.^2 , 3^ Ischemic strokes result from different vascular disorders including atherosclerosis, fibromuscular dysplasia, inflammatory and septic disorders, and arterial stenosis following calcified carotid artery atheroma (CCAA).

Cerebrovascular accident (CVA) is the third cause of death in the U.S, preceded only by cardiovascular disease and cancer. It is also the leading cause of severe disability, because 60% of the patients, who survive a stroke, suffer long-term physical and psychological disability.^3 , 4^ Therefore, atherosclerosis and its more dramatic consequences—stroke—represent a significant public health issue worldwide. In view of such a significant problem, every effort should be made to reduce the prevalence of CVA, including early detection of patients with CCAA.^5^

Although angiography is the gold standard for detecting CCAA and carotid stenosis, it is an invasive technique which requires specialized hospital equipments.^6^ On the other hand, Doppler sonography (DS) is widely used as an inexpensive, available, accurate and non-invasive technique for assessing carotid artery stenosis. While magnetic resonance angiography and computed tomography angiography continue to improve, color Doppler sonography provides information on the nature of the plaque, which is not available in these techniques. Although DS remains the current method for the diagnosis of carotid artery stenosis, its use for screening a large symptom-free population for the purpose of early diagnosis is not cost-effective.^7^ A more feasible option is to identify patients that have a higher probability of CCAA and refer them for DS.

Panoramic radiograph is a routinely-ordered screening method for almost all patients at referral to a dentist. Along with the evaluation of dental and maxillofacial hard tissues, panoramic radiograph can be used to spot soft tissue calcifications including CCAA. The known prevalence of positive incidental CCAA observed on panoramic radiographs taken for oral health reasons in the general dental outpatient population ranges from 2% to 5%.^7^ Although calcifications may not imply significant stenosis and not all atherosclerotic lesions are calcified, we hypothesized that the presence of calcifications seen on dental radiographs would be associated with significant carotid disease at a relatively high frequency that makes this finding a cost-effective incentive for the use of DS.

Thus, the current study was conducted to perform DS in asymptomatic dental patients with radiographically identified atheromas in panoramic views in order to prevent possible future strokes by the determination of hemodynamically significant lesions. We also assessed cardiovascular risk factors in such patients.

## Materials and Methods

In this cross-sectional study, a total of 960 panoramic radiographs of patients over 40 years (437 males and 523 females) were evaluated. All the patients had been referred to the Department of Oral and Maxillofacial Radiology, Mashhad University of Medical Sciences, during 2008-2009 for routine panoramic radiograph screening.

### Panoramic Radiography

**Figure 1:**
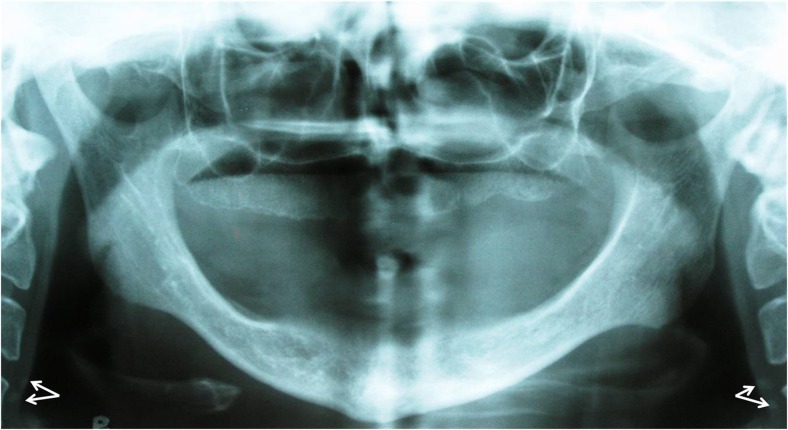
Panoramic radiograph of a 60-year-old woman shows bilateral small radiopacities of the carotid calcification (white arrows).

All radiographs were taken using PM 2002 CC (Planmeca, Helsinki, Finland). Patients’ head position and the exposure factors (kVp, mA) were specifically set for each individual. To obtain an identical density and contrast, we used Agfa Film/ORTHO, CP-G PLUS (AGPHA HealthCare, Mortsel, Belgium), Kodak Lanex regular screen (Eastman Kodak, New York, US), and Protect (PROTAC Medizintechnik GMbh&GO.KG, Germany) as the automatic film processor. To eliminate inter-examiner variability, one oral and maxillofacial radiologist examined all the radiographs. One or more heterogeneous radiopacities in a verticolinear orientation adjacent to the cervical vertebrae at or below the intervertebral space between C3 and C4 were considered as CCAA (Figure 1). Then the presence of CCAA on the left and right sides were separately recorded.

Patients were informed of the observations in their panoramic radiography. In case of consent, they were sent for DS.

### Doppler Sonography (DS)

Patients were referred to a general radiologist for DS using Sonix SP (Ultrasonix Medical Corporation, Richmond, Canada) with a linear probe of 10 MHz. The patients lied supine while their neck was slightly extended. The radiologist sited beside the patient’s thorax and scanned the neck. The radiologist also applied gel to the sonography area which usually allows access to the carotid even though it causes some degrees of resolution impairment. Sonography began with a transverse scan of the carotid artery in the neck area from the lowest possible border to the highest behind the angle of the mandible. This approach let the depth and course of the vessels be ascertained and areas of major disease be identified. Finally, DS was activated and the vessels were examined in the longitudinal plane.

**Figure 2:**
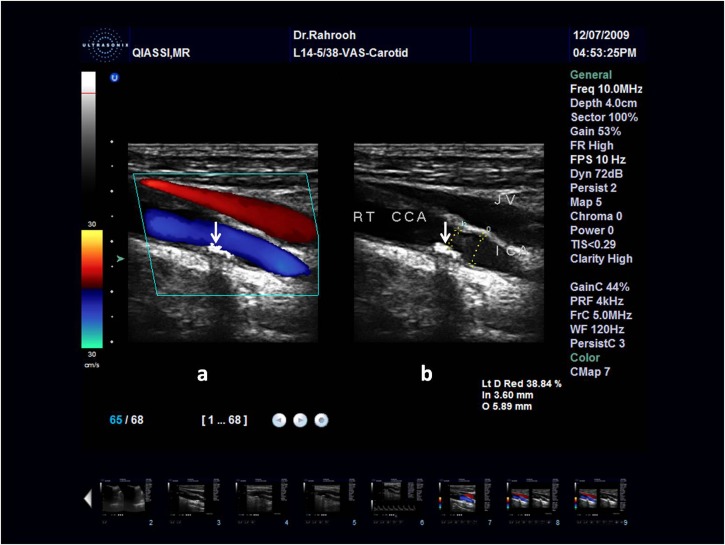
Longitudinal image of right common carotid and internal carotid artery of the same patient shows unilateral calcified plaque (white arrows) with 38.84% narrowing. (a) Color Doppler image. (b) Gray scale image: direct measurement of the caliber of the residual lumen and original caliber of the vessel.

Results of DS were set as:

1. Presence or absence of CCAA in each side.
2. Degree of the stenosis: as According to the International Asymptomatic Carotid Surgery Trial,^1^ endarterectomy of patients with more than 70% of stenosis and 75 years of age halves the risk of stroke during a period of 5 years. Therefore, we determined the degree of stenosis in order to recognize patients having at least 70% stenosis and direct them to a cardiovascular surgeon for further evaluations‎. Direct measurement of the caliber of residual lumen and original caliber of the vessel were used whenever the plaques were clearly visible, and color Doppler criteria were used whenever the region of plaque was invisible in order to determine the degree of the stenosis (Figure 2).^8 , 9^

The Patients completed a questionnaire including the history of cardiovascular attack, high blood pressure, diabetes mellitus, high cholesterol, smoking and obesity.

### Statistical Methods

The data were registered and analyzed using SPSS (Statistical Package for the Social Sciences, version 11.01; SPSS, Chicago, US)‎. The mean and standard deviation were calculated. Fisher test and independent t-test were employed for data analysis. P-values ≤ 0.05 were considered as statistical significance level.

## Results

In this study, 960 panoramic radiographs of patients over 40 years, including 524 females and 436 males, were evaluated. The mean age was 55.47 ± 10.56 years. Based on the radiographs, 19 (1.97%) patients (13 females and 6 males) had calcification. CCAA was more common in females (2.48%) than males (1.37%). Among the 19 patients, 15 (9 females and 6 males) admitted further evaluations which resulted in the examination of 30 sites on both left and right sides. 10 patients showed bilateral and 5 patients showed unilateral CCAAs in panoramic, including 11 sites on the right side and 14 on the left. In DS, 12 individuals had plaques on their carotid arteries in 16 sites (7 on the right side and 9 on the left; Table 1).

**Table 1:** Number (percent) of calcified carotid artery atheroma (CCAA) diagnoses in color Doppler sonography and panoramic radiography

Panoramic radiography	Color Doppler sonography	
	Positive	Negative
Positive	14 (46.66)	11 (36.66)
Negative	2 (6.66)	3 (10)
Total	16 (53.32)	14 (46.66)

Two patients showed stenosis greater than 70% (13.33%) and were referred to a cardiovascular surgeon.

Among several cardiovascular risk factors in the two groups of patients with CCAA and without CCAA, exact Fisher test revealed statistically significant relationship between age and presence of CCAA in DS (p=0.026). The comparisons of all risk factors including their frequencies are shown in Table 2.

**Table 2:** Comparison of frequency (percent) of risk factors in the two groups of patients with calcified carotid artery atheroma (CCAA) and without CCAA in color Doppler sonography

Risk factor	Patients with CCAA	Patients without CCAA	P-Value
Blood pressure	7 (58.33)	0 (0)	0.569
Diabetes mellitus	2 (16.66)	0 (0)	1
High cholesterol	2 (16.66)	1 (33.33)	1
Smoking	3 (25)	0 (0)	1
Obesity	7 (58.33)	1 (33.33)	0.282
Age	58.5^ a ^	43.33^ a ^	0.026*

^a ^Mean values have been reported for the age risk factor. * Statistically significant (p < 0.05)

## Discussion

In 1981, Arthur H. Friedlander displayed the possibility of identifying calcified atheroma plaques within the carotid artery on panoramic radiographs for the first time.^10^ In 1994, Friedlander and Baker^11^ reported an approximate frequency of 3% for carotid artery calcifications among a population of 295 individuals aged over 55 having no history of either a transient ischemic attack or CVA who were also asymptomatic‎. Also Carter et al^12^ reported a 3.6% prevalence for CCAA calcification in panoramic radiographs of patients who were subsequently referred for duplex ultrasound examination, which revealed no degree of stenosis requiring endarterctomy surgery. Ohba et al^13^ in Japan and Sismann et al^14^ in Turkey reported 5% and 5.06% prevalence of CCAA, respectively. Brand et al^15^ reported a 9.4% prevalence of asymptomatic CCAA in panoramic radiographs.

In the current study, the prevalence of calcification in panoramic radiographs of patients older than 40 years was 1.97%, which is lower compared to similar studies. This lower incidence may be due to genetic factors, nutritional status, age, number of patients, and gender. Furthermore, no difference was found between genders in presence of CCAA in color Doppler sonography. However, similar to Ohba et al^13^ and in contrast with Griniastsos et al,^16^ ‎ calcification as seen on panoramic radiograph was shown to be higher in females.^13 , 16^

Some studies have been conducted on the diagnostic accuracy of panoramic radiograph compared to more advanced techniques in detecting CCAA. Yoon et al^17^ compared panoramic radiography with CT as the gold standard, which resulted in the accuracy rate, sensitivity, and specificity of 62.3%, 22.2%, and 90.0% for panoramic radiography,‎ respectively. Furthermore, Damaskas et al^18^ showed that in detecting CCAA, panoramic radiography compared to DSA has lower sensitivity and specificity. However, a positive predictive value (PPV) of 100% for each patient and for luminal stenosis greater than 80% was documented.^18^

Moreover, several researchers have investigated the relationship between panoramic radiography and Doppler sonography. Ravon and Hollender^19^ reported that panoramic radiography has a higher PPV and negative predictive value (NPV) (≥ 79.3%) compared to Doppler sonography. Nevertheless, Bayram et al^20^ reported the PPV of 34.7%. Another study by Richard et al,^1^ showed a low sensitivity for panoramic radiography in detecting CCAA (31.1%) and stenosis (22.7%). Khosropanah et al^21^ also stated that panoramic radiography has a sensitivity of 45% and PPV of 66.6% compared to Doppler sonography in patients with coronary artery disease and a 50% of sensitivity in normal patients, based on which they reported the level of agreement as low.

In this study, we were supposed to identify asymptomatic patients at risk of CVA by the observation of CCAA in panoramic radiography as a screening method. We did not intend to determine the diagnostic accuracy of panoramic radiography in detecting CCAA, which requires another way of case selection, and also the application of panoramic radiography for patients who had CCAA in DS followed by the evaluation of sensivity, specifity, and predictive value for panoramic radiography.

Our DS results of the 25 sides with CCAA in panoramic demonstrated that 2 sides (13.33 %) had stenosis greater than 70%. However, Friedlander et al^22^ found 4 sides (4%) with at least 70 % stenosis. We believe that the discrepancies between the results of our study and those of the latter study‎ may have arisen from their use of different US criteria and possible selection bias in a small sample size.

In addition, many studies have evaluated the prevalence of CCAA in patients with systemic disorders including metabolic syndrome, dilated cardiomyopathy, diabetes mellitus type 2, obstructive sleep apnea or under treatment with therapeutic irradiation.^23 - 27^ All these patients have shown higher prevalence of CCAA in comparison with normal population, which may be due to underlying disorders causing atherosclerosis. Takeshi et al^13^ evaluated the relationship between CCAA detected in panoramic radiography and blood pressure, electrocardiography, blood cholesterol, fasting blood sugar, tooth number, and community periodontal index (CPI). As a result, a minor relationship was found between CCAA and general and oral health.^13^ Bayram et al^20^ also found no significant relationship between cardiovascular disease and coronary risk factors with CCAA.^20^ Moreover, Griniastsos et al^16^ reported a low prevalence of diabetes mellitus and hyperlipidemia in patients with CCAA based on their panoramic radiographs.^16^

In our study, the risk factors of cardiovascular diseases including blood pressure and cardiac disorders, high cholesterol, smoking, and obesity showed no statistically significant differences between the two groups of patients with CCAA and without CCAA in DS; however, a significant difference with age observed (p=0.026). This implies the importance of referring elderly patients with documents of calcification on their panoramic radiographs for further healthcare investigations.

With regard to the facts that CCAA is a major risk factor of CVA and can be detected on panoramic radiographs, the dentist has an important role in recognizing asymptomatic patients who are at a high risk for stroke.

Dentists should be educated about detection of CCAA in the panoramic radiographs and made aware of the importance of referring such patients—especially elderly ones—to physicians for complementary medical evaluations including DS, which could cause a decline in both morbidity and mortality rates of cerebrovascular attacks.
